# SOX10 modulated SMARCA4 dysregulation alleviates DNA replication stress in cutaneous melanoma

**DOI:** 10.1111/jcmm.17607

**Published:** 2022-11-01

**Authors:** Xiangjian Fang, Keqiang Rao, Zhiyi Wei, Juntao Cheng

**Affiliations:** ^1^ Department of Burns and Plastic Surgery Fujian Medical University Affiliated First Quanzhou Hospital Quanzhou Fujian Province China; ^2^ School of Medicine Shanghai Jiao Tong University Shanghai China

**Keywords:** bioinformatic, melanoma, replication DNA stress, SMARCA4, transcriptional modulation

## Abstract

Cutaneous melanoma (CM) is the most fatal type of skin cancer with a high potency of metastasis, yet the treatment for metastatic melanoma remains limited. In this study, we are devoted to addressing the prognostic value and underlying mechanism of DNA damage repair‐related genes in CM. We utilized integrated bioinformatic approaches and machine learning models to identify a cluster of convergently expressed DNA damage repair‐related genes in melanoma. With multivariate Cox regression, SMARCA4 (also known as BRG1) was identified as an independent prognostic marker for melanoma patients. Yet the expression of SMARCA4 is not altered with the pathological staging or the metastasis condition. SMARCA4 is an essential ATPase subunit of the mammalian SWI/SNF complex. Mechanistically, we demonstrated that SMARCA4 could resolve DNA replication stress and guarantee the proliferation of melanoma cells. Furthermore, we predicted the binding of different transcription factors on the SMARCA4 promoter and unveiled the modulated expression of SMARCA4 by SOX10 in melanoma. Together, we performed integrated approaches to identify SMARCA4 as a promising prognostic marker for melanoma, which was transcriptionally regulated by SOX10 and promoted melanoma cell proliferation by ameliorating DNA replication stress.

## 1 BACKGROUND

Arising from the melanocytes, cutaneous melanoma is the most fatal type of malignant skin neoplasm,^1^, ^2^ accounting for 75% of death cases in skin cancer. Meanwhile, as one of the most rapidly increasing types of cancer worldwide, melanoma cases were predominantly diagnosed in western populations.^3^ Early staged melanoma is a curable disease with surgical interventions.^4^ However, due to the lack of specific and sensitive diagnostic markers, metastasis was common in melanoma patients and their prognosis decreases dramatically.^5^ Previous studies have unveiled the critical role of exosome,^6^ AKT pathway^7^ and immunology alterations^8^ in the progression of melanoma, yet effective approaches for melanoma, especially for late‐staged patients, are still lacking. Therefore, screening for potent prognostic markers and druggable targets for melanoma is urgent.

Reactive oxygen species (ROS) is primarily produced in mitochondria and is the most prevalent cause of oxidative DNA damage in cells.^9^ Specifically, ROS features a high affinity to guanine and produces 8‐oxo‐dG, the most predominant form of oxidized nucleotides in cells.^10^ Single‐stranded DNA was exposed during the S phase and becomes highly vulnerable to ROS,^11^ leading to strand breaks in genome DNA.^12^ And the unrepaired DNA strand breaks would activate different cell cycle checkpoints and cause cell cycle arrest.^13^, ^14^ Therefore, cells are highly dependent on the DNA damage repair pathways to adequately deal with oxidized lesions and to maintain proliferation, especially in cancer cells.

Cancer cells harbour frequent mutations in DNA damage repair‐related genes with high proliferation potential, which convergently causes the accumulation of oxidized lesions and strand breaks in the genomic DNA.^15^, ^16^ To survive such replication stress, multiple DNA damage repair pathways are persistently activated during the S phase, especially with diverse gain‐of‐function mutations.^17^ Specifically, melanoma cells feature activated DNA damage repair pathways, among which homologous recombination pathway (HR) and nucleotide excision repair pathway (NER) are typically activated during DNA replication.^18^, ^19^, ^20^ Also, the mutations in the NER pathway could cause the collision of the replication fork and the termination of DNA replication during the S phase.^18^, ^21^ Yet the detailed mechanism unveiling how NER pathway genes could prevent DNA replication stress and contribute to the proliferation of melanoma remains poorly understood.

SMARCA4 (also known as BRG1) in the SWI1/SNF1 family is the central component of the SWI1/SNF1 complex with high helicase/ATPase activity.^22^, ^23^ The alteration of the SWI/ SNF complex is generally considered the centre of chromatin remodelling and epigenetic modifications.^22^ Besides, SMARCA4 is reported to be involved in the transcription of multiple downstream genes, such as EGFR signalling in colon cancer^24^ and SREBP1c in lipid metabolism.^25^ SMARCA4 also regulates gene expression^26^ by binding with H3K27me3 modifiers and suppresses transcription‐associated genomic instability by recruiting TOP1.^27^ More importantly, SMARCA4 could increase chromatin accessibility and promote the repair of double‐strand DNA damage via the NER pathway^28^ and participate in the solution of R‐loop‐mediated transcription–replication conflicts in cells.^29^ However, the crucial role of SMARCA4 in the elimination of replication stress in melanoma remains elusive.

## 2 MATERIAL AND METHOD

### 2.1 Data collection

mRNA array data, DNA methylation data and clinical data of melanoma patients and other cancer types were acquired from the TCGA SKCM database (https://tcga‐data.nci.nih.gov/) and 3 GEO database (GSE22155, GSE50509 and GSE65904, https://www.ncbi.nlm.nih.gov). PAN‐Cancer expression patterns of SMARCA4 are acquired from the GEPIA database (http://gepia.cancer‐pku.cn/index.html). Gene expression of SMARCA4 in normal skin and Skin cutaneous melanoma were compared with the OncoDB tool (http://oncodb.org/).

### 2.2 SC3 (Single Cell Consensus Clustering) unsupervised clustering

Single‐cell consensus clustering (SC3) was carried out with the mRNA array data from the TCGA SKCM database. The corresponding script was acquired from Bioconductor (http://www.bioconductor.org/) and modified by the author.

The dimension reduction with the tSNE method was achived with python and the script was acquired and modified from CSDN (https://blog.csdn.net/).

### 2.3 Network analysis and Procrustes analysis

The network analysis and Procrustes analysis were performed on the Tutools platform (https://www.cloudtutu.com/).

Network analysis and Procrustes analysis were performed to analyse the correlation within a cluster of genes or compare the co‐occurrence patterns between mRNA and copy number variation in the TCGA SKCM database.

### 2.4 Kaplan–Meier analysis

Kaplan–Meier analysis was performed in this study to compare the overall survival (OS), disease‐specific survival (DSS) and disease‐free interval (DFI) in melanoma patients.

Kaplan–Meier analysis was performed with GraphPad (https://www.graphpad.com/) to calculate the Hazard ratio and log‐rank significance in different groups of melanoma patients. Besides, Kaplan–Meier analysis was performed with SPSS to stratify patients concerning metastasis status in melanoma patients.

The cut‐off value for all the Kaplan–Meier analyses in this article is defined by the median of its corresponding expression level. Gene expression higher than the cut‐off value is defined as the high‐expression group and vice versa.

### 2.5 Multiple variate Cox regression

Multiple variate Cox regression was performed with SPSS software (SPSS 22.0, https://www.ibm.com/cn‐zh/analytics/spss‐statistics‐software) to select independent factors for the prognosis of melanoma patients. The forward stepwise method was used in the multivariate Cox Regression model with *p* < 0.05 to enter and *p* < 0.10 to exit.

Independent factors which are significant in predicting OS, DSS and DFI were selected and the risk score for OS and DFI were separately established with the multivariate Cox regression model in the melanoma patients.

### 2.6 Heatmap and hierarchical clustering

Heatmap with hierarchical clustering was achieved with R software (https://www.r‐project.org/). For Heatmap, the expression level of each gene was normalized to the median in each patient and the colour scale was normalized as indicated. Hierarchical clustering was performed with the Euclidean clustering method with complete linkage and optimized gene/sample order. For heatmaps describing the Pearson correlation between two genes, the Euclidean clustering was directly performed without normalization.

Heatmap and the following hierarchical clustering enabled us to directly visualize and comprehend the expression pattern of a certain cluster of genes or samples.

### 2.7 Correlation analysis and student's *t*‐test

Correlation between two groups of samples (including Figures 2H and 5C, Figures S3F and S5D) was performed with SPSS and plotted with GraphPad. Linear regression was performed with GraphPad with 95% CI labelled.

Student's *t*‐test (including Figures 3D–E, 4A,B,D,F–G, 5D–G, Figures S1C–E and S3B–E) was performed with GraphPad.

### 2.8 Prediction of the transcriptional modulation of SMARCA4

To further explore the potential transcriptional modulation of SMARCA4 in melanoma patients, we first acquired the DNA sequence of the SMARCA4 promoter (−2000 bp to −1 bp) in the UCSC database (http://www.genome.ucsc.edu/index.html). Then, we predicted the binding affinity of all the known human transcription factors listed in the JASPAR database (http://jaspar.genereg.net/)and the PROMO database (http://alggen.lsi.upc.es/recerca/frame‐recerca.html).

Next, the transcription factors with at least 2 binding site <5% dissimilarly in the PROMO database and with a relative profile score >90% was selected as potential transcription factors for the modulation of SMARCA4. We then analysed the expression level of SMARCA4 and candidate transcription factors in 4 different databases with Pearson analysis.

### 2.9 Cell culture

Two melanoma cell lines, M14 and A375, were purchased from the National Collection of Authenticated Cell Culture in the Chinese Academy of Sciences. Cells were cultured in DMEM supplemented with 10% FBS and 1% penicillin and streptomycin at 37 degrees in a humidified chamber with 5% CO2.

### 2.10 Cell proliferation assay and clonogenic assay

For the proliferation assay, cells were seeded in the 96‐well plate at 2000 cells/well and cultured for 5 days. The proliferation curve was plotted with CCK8 (Cell Counting Kit‐8).

For the clonogenic assay, cells were depleted of SMARCA4 with specific siRNA transfection. 24 h after transfection, cells were re‐seeded into the 6‐well plate at 200 cells/well at triplicates and cultured for 14 days. Cells were stained with crystal violet and manually counted under the microscope.

### 2.11 siRNAs, Constructs and transfections

siRNAs sequences used in this study were reported earlier^24^, ^30^ and were purchased from Shanghai Genomeditech. siRNA transfection was performed with JetPRIME Polyplus (*Polyplus*114‐01) according to the manufacturer's instructions and the siRNA efficacy was measured 72 h post‐transfection.

siRNA sequences are as follows:siSMARCA4 s1: 5′‐ UCGCUUUG GUUCGCAAAUC3’.

siSMARCA4 s2: 5′‐ UUCCUCCUCAUUCAGGUCC‐3’.

siSOX10 s1: 5′‐ GAACGAAAGUGACAAGCGC‐3’.

siSOX10 s2: 5′‐ GCGGGAAGCCUCACAUCGA‐3′.

### 2.12 Antibody and reagents

The following primary antibodies were used:

**Table:** 

SMARCA4	PA5‐40697	Rb	Thermo	ChIP
γH2AX	9718 S	Rb	CST	IF

The following secondary antibodies were used:

**Table:** 

Alexa Fluor® 555 anti‐Rabbit	ab150078	Goat	Abcam	IF

### 2.13 Immunofluorescence and EdU staining

Immunofluorescence was performed as previously described.^30^ Briefly, cells were fixed with 4% PFA and permeabilization with 0.1% Triton, then cells were washed twice with PBS and blocked for 1 h in 3% BSA. Cells were incubated with indicated primary antibody (diluted in 3% BSA) and the corresponding secondary antibody, followed by washing with PBS. Finally, cells were counterstained with DAPI before image acquisition.

For the γH2AX‐EdU colocalization experiment, cells were first transfected with specific siRNAs for 72 hours before EdU (C0081S, Beyotime) was added to the cells. After incubation with 1:1000 EdU for 30 min, cells were fixed with 4% PFA, followed by cell permeabilization, blocking, and incubation with primary/secondary antibodies. Last, the EdU‐Click reaction is performed according to the manufacturer's protocol. After 5 times washing with PBS, cells were counterstained with DAPI, and images were acquired. The colocalization results were analysed with Cell profiler (https://cellprofiler.org/).

### 2.14 Chromatin immunoprecipitation (ChIP)

ChIP was performed with a Chromatin Immunoprecipitation kit (Merck Millipore, MA, USA) per the manufacturer's instructions. Briefly, cells were cultured in a 15 cm dish till 80% confluency and were crosslinked with 550ul 37% formaldehyde (1% final concentration) for 10 min at room temperature. Cells were harvested and the nuclear was isolated and resuspended in SCW buffer. After sonication on ice, samples were centrifuged and the supernatant was collected. At the same time, Magna ChIP Protein A/G Magnetic Beads were washed and incubated with SOX10 antibody (Thermo Fisher, PA5‐40697) or IgG control. Then, samples were immunoprecipitated with antibody‐labelled magnetic beads overnight. Samples were repeatedly washed and incubated with Elution Buffer with proteinase K.

Immunoprecipitated DNA was collected, and the enrichment of the DNA template was analysed by conventional quantitative PCR, using primers specific for SMARCA4 promoter (targeting the ‐586 bp to −489 bp region) and negative control primers (targeting the ‐431 bp to −331 bp region). Primers sequences were listed as follows:

ChIP_1442_F: 5′‐ TCCTTCCCCACTAGACCGAG −3’.

ChIP_1442_R: 5’‐GCAAAACTTCCCAAGTGCCA −3’.

ChIP_Neg_F: 5′‐ CAGGTCAGGGATCAAAGCGG −3’.

ChIP_Neg_R: 5′‐ TAGGAACCCTGGACCGTAGG −3′.

### 2.15 Quantitative polymerase chain reaction (qPCR)

Qiagen RNA isolation kit and reverse transcriptase were used to extract total RNA from tissues and cultured cells and to synthesize complementary DNA.

qPCR was performed using SYBR Premix Ex Taq (Takara) in a Bio‐Rad CFX‐96 Real‐Time PCR detector, using GAPDH as the internal control. The stability of GAPDH was examined in Figure S5 E. The Primers used in this study are as follows:

h_ GAPDH _F: 5′‐ GCCATGTAGACCCCTTGAAGAGC‐3′;

h_ GAPDH _R: 5′‐ ACTGGTTGAGCACAGGGTACTTTAT‐3′.

h_ SMARCA4 _F: 5′‐ CAGATCCGTCACAGGCAAAAT −3′;

h_ SMARCA4 _R: 5′‐ TCTCGATCCGCTCGTTCTCTT −3′.

### 2.16 Dual‐luciferase reporter assay

Dual‐luciferase reporter assay was performed according to the manufacturer's protocol (Beyotime, RG027). Briefly, cells were co‐transfected with firefly luciferase control plasmid along with renilla reporter plasmid at a ratio of 10:1. After 48 h, cells were harvested and lysed in the lysis buffer. The activity of luciferase was detected by the Dual‐Luciferase Reporter Assay System. The results were normalized to the renilla activities.

SMARCA4 promoter regions (−600 bp to −300 bp) used in the study:

GTCCTCCTTCCCCACTAGACCGAGGGCTCCACGGCGGCTGGG**ACACAG**TAGCAGATCAATAAATATTTGCTAAGCTAATTAATGGCACTTGGGAAGTTTTGCAGAGAAGGCGGCCACGGTCGGGCCCCGCCTTGCCTCCCCAAATAGGCCTCGCCGCCCCAGGTCAGGGATCAAAGCGGTTCCCAGGCGCGCCCTTGGCCCGCGGGAAACCACTGCCCGGTCTTGGTCCAGGCGGCCCGTCCTACGGTCCAGGGTTCCTATTTCCGAGCCTCAGGGACCTCCTTTCCCCACGGACCCCAC.

Binding domain depleted SMARCA4 promoter (−600 bp to −300 bp) used in the study:

GTCCTCCTTCCCCACTAGACCGAGGGCTCCACGGCGGCTGGGTAGCAGATCAATAAATATTTGCTAAGCTAATTAATGGCACTTGGGAAGTTTTGCAGAGAAGGCGGCCACGGTCGGGCCCCGCCTTGCCTCCCCAAATAGGCCTCGCCGCCCCAGGTCAGGGATCAAAGCGGTTCCCAGGCGCGCCCTTGGCCCGCGGGAAACCACTGCCCGGTCTTGGTCCAGGCGGCCCGTCCTACGGTCCAGGGTTCCTATTTCCGAGCCTCAGGGACCTCCTTTCCCCACGGACCCCAC.

## 3 RESULTS

### 3.1 Melanoma patients harbour distinct patterns of DNA damage repair‐related genes

**FIGURE 1:**
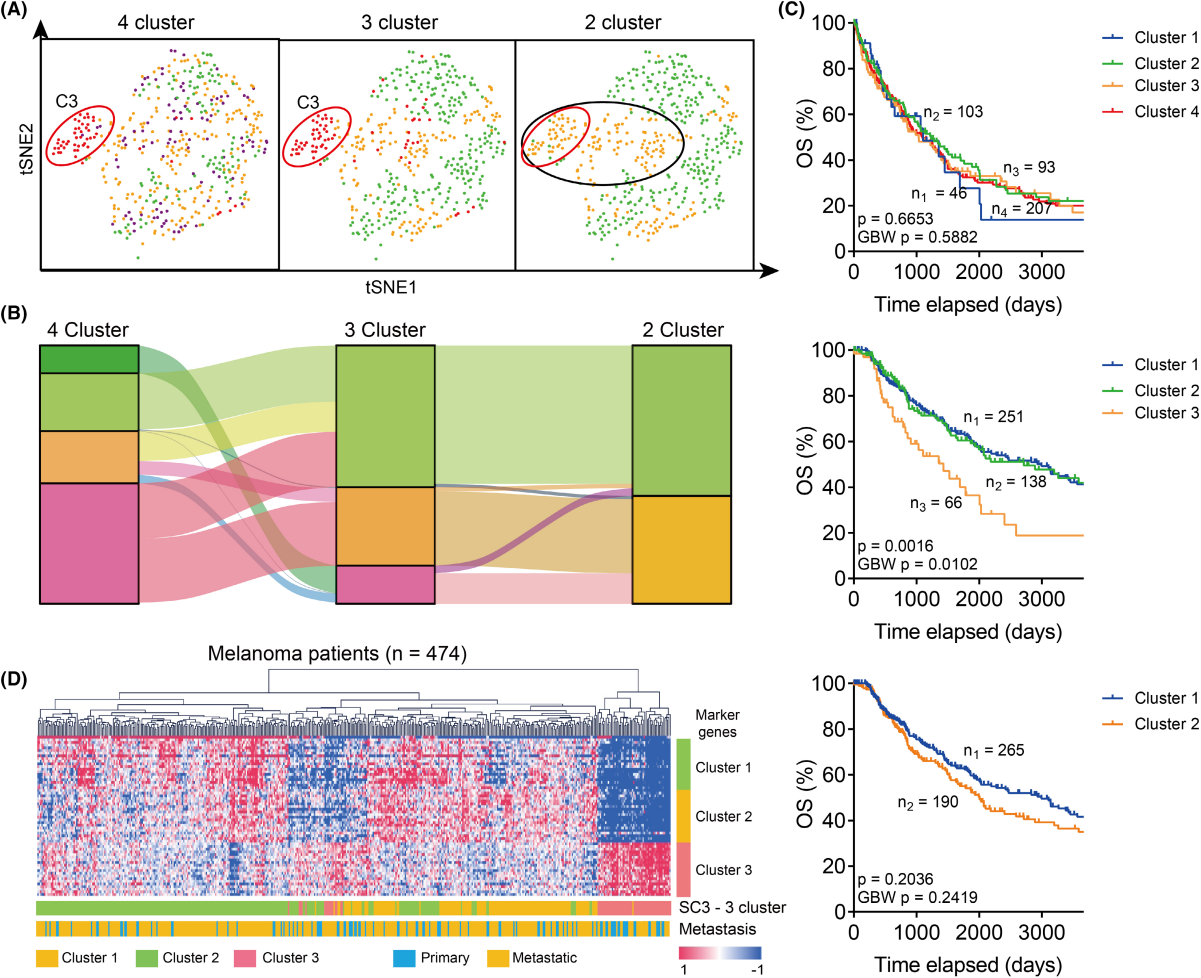
Melanoma patients harbour distinct patterns of DNA damage repair‐related genes. (A). Results of Single Cell Consensus Clustering (SC3) in melanoma patients. Patients were divided into 2, 3, or 4 clusters, and different clusters were labelled in different colours. (B). Sanky plot showing the relationship between 2, 3, or 4 clusters of melanoma patients. (C). Kaplan–Meier analysis comparing the overall survival (OS) of different clusters of melanoma patients in A. (D). Heatmap and Euclidean clustering of melanoma patients in TCGA SKCM database with complete linkage. Different clusters of patients, metastasis status of patients and the corresponding marker genes were shown.

To comprehend how DNA damage participates in the remodelling of distinct features of melanoma cells, we performed unsupervised clustering with all DNA damage repair‐related genes in melanoma patients from the TCGA database (Figure 1A). And melanoma patients were categorized into 2 to 4 clusters with the single cell consensus clustering (SC3) method and patients showed better consistency when clustered into 3 groups (Figure 1A–B). In concert with this, we noticed only Cluster 3 showed significantly reduced overall survival when divided into 3 groups (Figure 1C). Also, both the disease‐specific survival (DSS) and the disease‐free interval (DFI) were reduced in Cluster 3 patients (Figure S1A–B).

We further compared the expression level of DNA damage repair genes and the clinical features of Cluster 3 melanoma patients. We noticed that Cluster 3 patients were synergistically overexpressed in a cluster of DNA damage repair‐related genes, defined as Cluster 3 marker genes. However, there is no significant difference in the metastasis condition (Figure 1D), age (Figure S1C), Breslow depth value (Figure S1D) and mitotic cell counts (Figure S1E). Also, the distribution of pathological staging (Figure S1F), radiation therapy condition, pharmaceutical therapy condition, neoadjuvant treatment condition, additional surgery condition (Figure S1G), Clark value level (Figure S1H) or the types of new tumour events (Figure S1I) were not altered in Cluster 3 melanoma patients.

### 3.2 Identification of prognostic markers in Cluster 3 marker genes in melanoma

**FIGURE 2:**
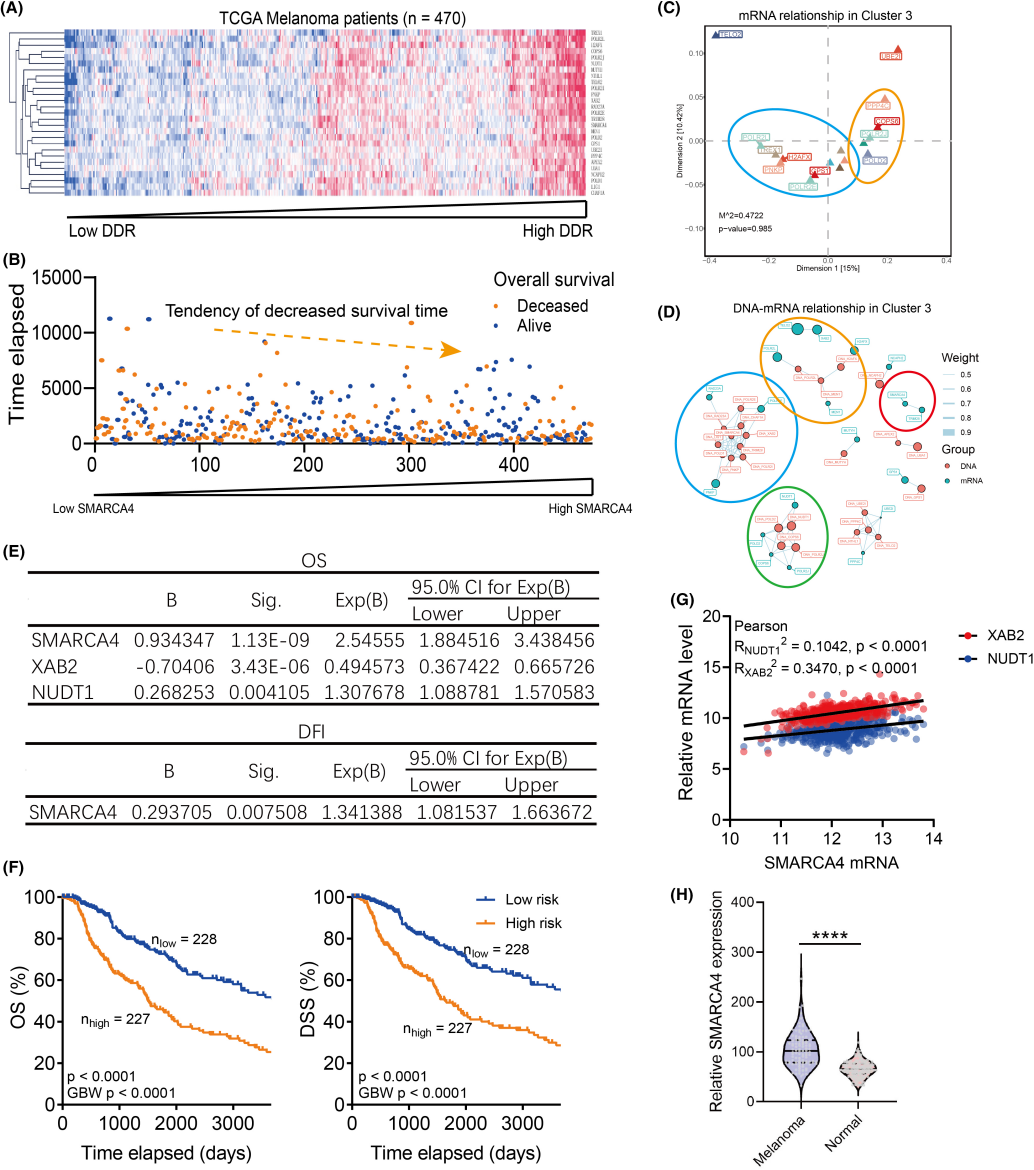
Identification of prognostic markers in Cluster 3 marker genes in melanoma. (A). Heatmap and Euclidean clustering with complete linkage showing the expression of top 38 marker genes for Cluster 3 in melanoma patients. (B). Dot plot showing the prognosis of melanoma patients with the increase of Cluster 3 gene expression. The tendency of survival time was plotted with linear regression. (C). Procrustes analysis showing the relationship between differentially expressed genes in C in TCGA SKCM and GSE22115 databases. (D). Network analysis showing the relationship between the mRNA and the copy number variation of differentially expressed genes in C in the TCGA SKCM database. (E). Multivariate Cox regression model with the forward stepwise method showing the predicted risk factors for overall survival (OS, the upper panel) and disease‐free interval (DFI, the lower panel). (F). Kaplan–Meier analysis comparing the OS (the left panel) and DSS (the right panel) concerning the level of the risk score calculated as in E. (G). Dot plot showing the correlation between SMARCA4 and XAB2 or NUDT1 in melanoma patients in the TCGA SKCM database. Pearson correlation R square was shown. (H). Violin plot showing the SMARCA4 mRNA level in melanoma and normal skin tissues.

We further plotted the expression level of Cluster 3 marker genes in melanoma patients and selected the top 38 synergistically expressed genes with Pearson correlation >0.3 (Figure S2A–B). Higher DNA damage repair level was closely correlated with the prognosis of melanoma patients (Figure 2A–B and Figure S2C–D) and differentially expressed Cluster 3 marker genes were selected (Figure S2 E). Differentially expressed Cluster 3 marker genes showed similar expression patterns (Figure 2C) and consistency to copy number variation (Figure 2D).

A multivariate Cox regression model was used to avoid bias from the co‐linearity of Cluster 3 marker genes. The result showed that SMARCA4, XAB2 and NUDT1 were potent markers for the overall survival (OS) and disease‐specific survival (DSS) (Figure 2E–F) and SMARCA4 was shown to correlate with XAB2 and NUDT1 expression in melanoma patients (Figure 2G). Besides, SMARCA4 was a promising prognostic marker for the disease‐free interval (DFI) in melanoma patients (Figure S2 E) and was significantly elevated in melanoma patients, compared with normal skin tissues (Figure 2H).

### 3.3 SMARCA4 correlates with the proliferation potential of melanoma patients

**FIGURE 3:**
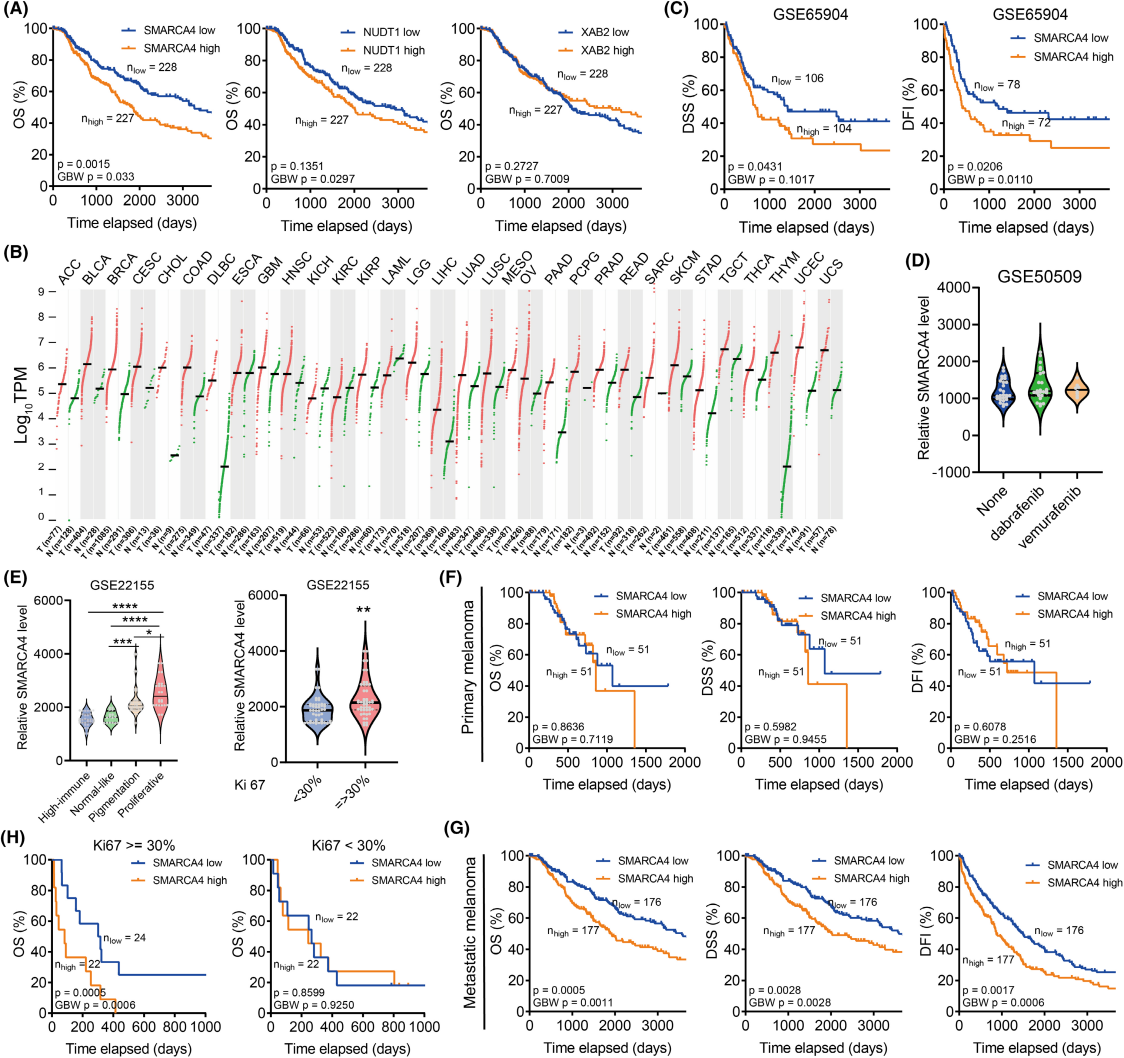
SMARCA4 correlates with the proliferation potential of melanoma patients. (A). Kaplan–Meier analysis comparing the survival of different levels of SMARCA4 (the left panel), NUDT1 (the middle panel) or XAB2 (the right panel) in melanoma patients. (B). The expression profile of SMARCA4 in 22 types of cancers in the TCGA database. Cancer tissues were shown in red and the non‐cancer tissues were shown in green. (C). Kaplan–Meier analysis comparing the disease‐specific survival (DSS) or disease‐free interval (DFI) of different levels of SMARCA4 in the GSE65904 database. (D). The expression pattern of SMARCA4 in dabrafenib or vemurafenib treated melanoma patients in the GSE50509 database. (E). The expression of SMARCA4 in melanoma patients with different categories (the left panel: high immune, normal like pigmentation or proliferative group) or different ki67 levels (the right panel, <30% or >=30%). (F–G). Kaplan–Meier analysis comparing the overall survival of primary melanoma (F) or metastatic melanoma (G) with different SMARCA4 levels. (H). Kaplan–Meier analysis comparing the overall survival of high proliferative melanoma (the left panel, ki67 >=30%) or low proliferative melanoma (the right panel, ki67 < 30%) with different SMARCA4 levels. Data were represented as mean ± SEM. **p < 0.01. The data were analysed using Student's *t*‐test.

To decipher potent prognostic markers for melanoma, we performed the Kaplan–Meier analysis and observed only SMARCA4 could accurately predict the unfavourable prognosis of melanoma patients (Figure 3A and Figure S3A). Therefore, we were devoted to unveiling the prognostic value and the biological function of SMARCA4 in melanoma.

SMARCA4 is generally overexpressed in multiple cancer types compared to the non‐tumour control (Figure 3B) and the predicted unfavourable outcome in the GEO database (GSE65904, Figure 3C). The expression of SMARCA4 is not altered after dabrafenib or vemurafenib treatment in melanoma patients (Figure 3D) and is highly increased in proliferative melanoma and ki67 positive (>30%) melanoma (Figure 3 E). But the expression level of SMARCA4 is not changed with the BRAF status, TNM staging, Clark value level, metastasis status or Breslow depth value (Figure S3B–F).

Further analysis revealed that SMARCA4 especially correlated with favourable prognosis in metastatic and highly proliferative melanoma patients, but not in primary melanoma patients (Figure 3F–H). Also, SMARCA4 mutation and reduced SMARCA4 copy number predicted favourable prognosis in melanoma patients (Figure S4A–B).

### 3.4 SMARCA4 resolves DNA replication stress in melanoma cells

**FIGURE 4:**
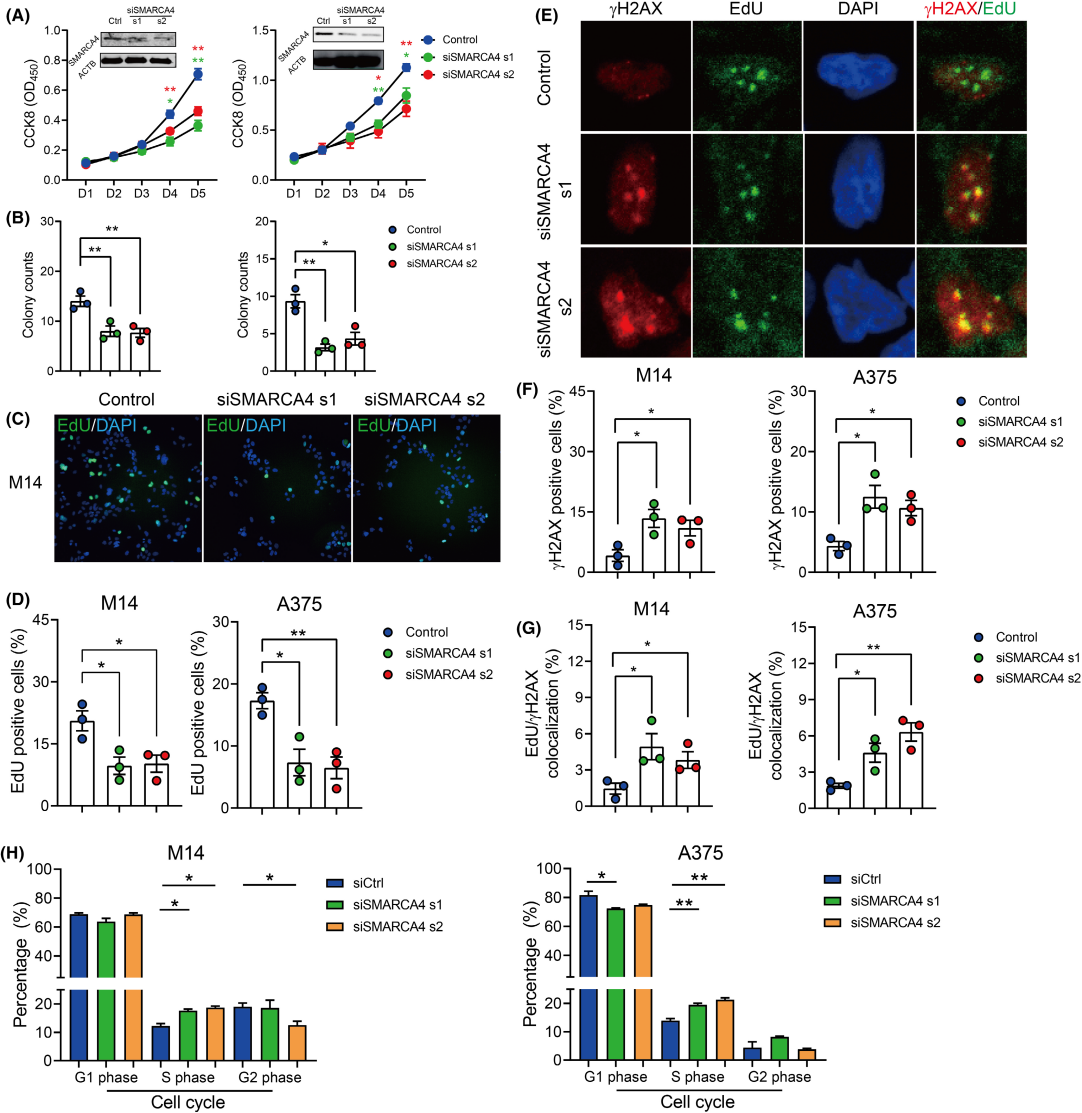
SMARCA4 resolves DNA replication stress in melanoma cells. (A). Proliferation curve of SMARCA4 depleted M14 (the left panel) and A375 (the right panel) cells with CCK8. (B). Colony counts in SMARCA4 depleted M14 (the left panel) and A375 (the right panel) cells with clonogenic experiments. (C‐D). Typical figures (C) and quantification (D) of EdU positive cells in SMARCA4 depleted M14 (D, the left panel) and A375 (D, the right panel) cells. EdU (green) was detected with EdU‐Click reaction. (E‐G). Typical figures (E) and quantification of γH2AX foci (F) or EdU‐γH2AX colocalization (G) in SMARCA4 depleted M14 (F‐G, the left panel) and A375 (F‐G, the right panel) cells. γH2AX was stained with immunofluorescence (red) and EdU was stained with EdU‐Click reaction (green). (H). Quantification of flow cytometry data comparing the G1, S and G2 phase cells in SMARCA4 depleted M14 and A375 cells. Data were represented as mean ± SEM. *p < 0.05; **p < 0.01. The data were analysed using Student's *t*‐test. All experiments were repeated at least 3 times.

To better elucidate the biological function of SMARCA4 in melanoma, we examined the proliferation ability and the clonogenic ability of M14 and A375 cells. The result showed reduced proliferation and clonogenic ability in SMARCA4‐depleted melanoma cells (Figure 4A–B).

Replication stress features increased DNA replication damage, cell cycle arrest (typically in the S phase) and increased apoptosis in cells. To explore the underlying mechanism of SMARCA4, the DNA replication fork was labelled with EdU, and DNA damage sites were labelled with γH2AX in cells. As a result, SMARCA4‐depleted M14 and A375 cells showed significantly reduced replication (Figure 4C–D) and increased DNA damage by γH2AX staining (Figure 4 E–F). Increased colocalization of EdU and γH2AX was observed in SMARCA4 depleted cells, suggesting increased DNA damage at the replication site (Figure 4 E,G). Flow cytometry results showed a significant accumulation of S phase cells (Figure 4H), but not necrotic/apoptotic cells after SMARCA4 depletion (Figure S4C).

### 3.5 SOX10 transcriptionally regulates SMARCA44 expression in melanoma

SMARCA4 gene was highly methylated in the TCGA database (Figure S5A) and cg08151828, cg26967868 and cg23963476 showed the highest correlation with SMARCA4 mRNA level (Figure S5B), among which cg23963476 is negatively correlated with SMARCA4 and correlates with the survival of melanoma patients (Figure S5C–D).

**FIGURE 5:**
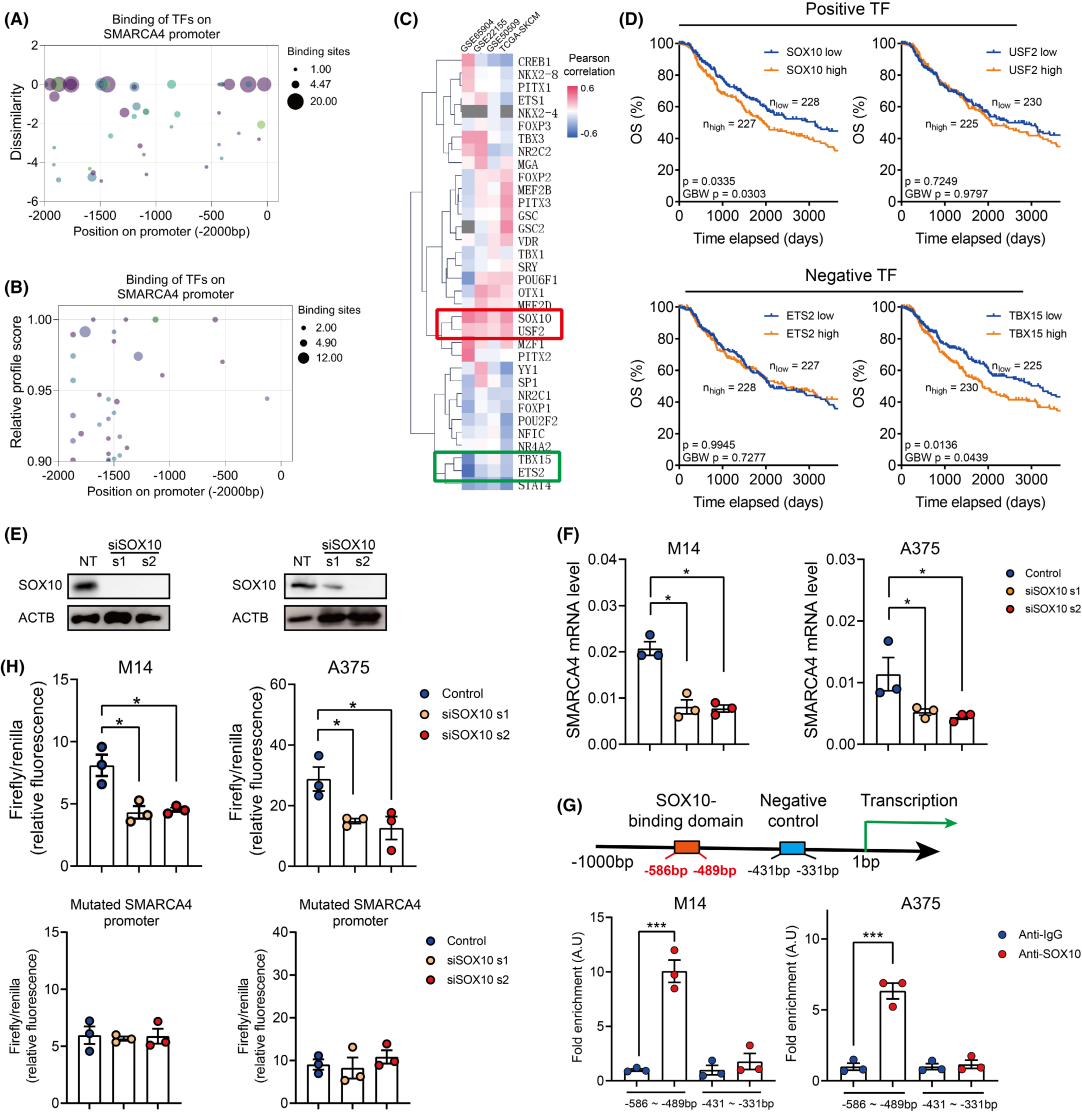
SOX10 transcriptionally regulates SMARCA4 expression in melanoma. (A–B). Bubble plot showing the binding of transcription factors on the SMARCA4 promoter in PROMO database (A) and JASPAR database (B). (C). Heatmap showing the Pearson correlation between mutual transcription factors in A–B and SMARCA4 in the TCGA SKCM database. (D). Kaplan–Meier analysis comparing the overall survival of melanoma patients with different levels of 2 positive transcription factors (SOX10, USF2; the upper panel) and 2 negative transcription factors (ETS2 and TBX15; the bottom panel). (E). Immunoblots showing the SOX10 level in SOX10 depleted M14 (the left panel) and A375 cells (the right panel). (F). Quantification of SMARCA4 mRNA levels in SOX10 depleted M14 (the top panel) or A375 (the bottom panel) with qPCR. (G). Upper panel: schematic figure showing the binding site of SOX10 at the SMARCA4 promoter region and the negative control region for ChIP‐qPCR. Bottom panel: quantification of ChIP‐qPCR experiment comparing the fold enrichment of SOX10 on the promoter region of SMARCA4 in M14 (the left panel) or A375 (the right panel) cells. (H). Quantification of dual luciferase assay comparing the transcription activity ofSMARCA4 promoter (the upper panel) or SOX10 binding domain‐depleted SMARCA4 promoter (the bottom panel) in SOX10 depleted M14 or A375 cells. Data were represented as mean ± SEM. *p < 0.05; ***p < 0.001; ****p < 0.0001. The data were analysed using Student's *t*‐test. All experiments were repeated at least 3 times.

To further explore the upstream regulation of SMARCA4, we predicted the binding of all the listed transcription factors in the PROMO database and JASPAR database. Transcription factors with <5% in dissimilarity or >90% in relative profile score were shown in Figure 5A–B. Then, we analysed the correlation between the expression level of SMARCA4 and separate transcription factors in the TCGA database with Pearson regression. Pearson regression results from 4 different melanoma databases showed that SOX10 and USF2 were positively correlated with the expression of SMARCA4, yet TBX15 and ETS2 were negatively correlated with the expression of SMARCA4 (Figure 5C). Both SOX10 and TBX15 were correlated with the unfavourable prognosis of melanoma patients, and SOX10 positively correlates with SMARCA4 (Figure 5D).

SOX10 is a well‐established transcription factor, whose function has been discussed in multiple cancer types^31^, ^32^ and is critical for the proliferation of melanoma.^33^ Meanwhile, SMARCA4 was reported to regulate SOX10 expression or function as a co‐activator of SOX10 in melanoma.^34^, ^35^, ^36^ Here, we hypothesized that SOX10 could transcriptionally modulate SMARCA4 expression and maintain melanoma cell proliferation. SOX10 expression was depleted with specific siRNAs and the transcription of SMARCA4 was dramatically suppressed (Figure 5 E–F). Also, the ChIP experiment in M14 and A375 cells suggested that SOX10 could specifically bind to the promoter region of SMARCA4 (Figure 5G). Consistently, dual‐luciferase reporter assay showed the transcription of SMARCA4 is greatly suppressed by SOX10 depletion (Figure 5H).

## 4 DISCUSSION

Melanoma is a fatal skin malignancy prevalently diagnosed in western populations.^1^, ^2^ So far, the treatment for metastatic melanoma is limited and the prognosis of melanoma patients remains unsatisfactory.^37^ The pathological function of multiple DNA damage repair‐related genes, especially the NER‐related genes, in the carcinogenesis and the progression of melanoma has been suggested,^38^ yet how SMARCA4 promotes the proliferation of melanoma cells has not been discussed.

With unsupervised clustering with machine learning, we managed to divide melanoma patients into different clusters. Interestingly, we observed that the Cluster 3 patients showed the worst prognosis, independent of the metastasis status of melanoma patients (Figure 1 and Figure S1). By looking into the expression pattern of marker genes in Cluster 3 patients, we set up a multivariate Cox regression model and selected SMARCA4 as the potential prognostic marker for melanoma (Figure 2 and Figure S2).

The expression of SMARCA4 was elevated with the proliferation potential of melanoma and effectively predicted the prognosis of metastatic and proliferative melanoma patients (Figure 3 and Figure S3). Consistently, somatic mutations and decreased copy number of SMARCA4 predicted a tendency towards the favourable outcome of melanoma patients (Figure S4), indicating SMARCA4 was involved in the proliferation of melanoma.

To further explore the function of SMARCA4, we compared the proliferation and clonogenic ability of SMARCA4‐depleted melanoma cells. As a result, we showed significantly increased DNA replication damage and suppressed DNA replication after SMARCA4 depletion (Figure 4), which suggests that SMARCA4 functions to eliminate DNA replication damage and maintains cell proliferation.

To explore the potential upstream regulation network of SMARCA4, we predicted the binding affinity of all the potential transcription factors with the promoter of SMARCA4 in the PROMO and JASPAR database. And SOX10 was predicted to bind to the promoter region of SMARCA4 and correlated with SMARCA4 across 4 different databases. Interestingly, multiple studies have reported that SMARCA4 could directly modulate SOX10 expression^34^, ^36^ or function as a co‐activator of SOX10 in melanoma.^35^ However, whether SOX10 could directly modulate SMARCA4 transcription remains unknown. Eventually, the binding of SOX10 at the promoter of SMARCA4 was validated with ChIP‐qPCR and its effect in promoting SMARCA4 transcription was validated with dual luciferase assay (Figure 5).

Together, we described the overall landscape of DNA damage repair in melanoma and clustered melanoma patients with distinct prognoses. We demonstrated the expression pattern and the clinical relevance of SMARCA4 and unveiled its physiological function in resolving DNA replication damage. Lastly, we predicted and validated the potential transcription factors for SMC4 expression. And the limitation of the study is that the study was largely based on computational results. Therefore, further exploration into the mechanisms and further validation with in vitro/in vivo models and melanoma patients is needed. While it seems a long way to find a promising approach for melanoma, we believe SMARCA4 could be a promising target for the treatment of melanoma patients.

## AUTHOR CONTRIBUTIONS

**Xiangjian Fang:** Conceptualization (equal); data curation (equal); formal analysis (equal); investigation (equal); methodology (equal); validation (equal); visualization (equal); writing – original draft (equal); writing – review and editing (equal). **Zhiyi Wei:** Conceptualization (equal); formal analysis (equal); investigation (equal); methodology (equal); visualization (equal); writing – original draft (equal); writing – review and editing (equal). **Juntao Cheng:** Conceptualization (equal); data curation (equal); formal analysis (equal); project administration (equal); supervision (equal); validation (equal); writing – original draft (equal); writing – review and editing (equal). **Keqiang Rao:** Formal analysis (equal); methodology (equal); writing – review and editing (equal).

## CONFLICT OF INTEREST

The authors declare that they have no competing interests.

## Supporting information


Figure S1

## Data Availability

The datasets generated and/or analysed during the current study are available in the TCGA SKCM database (https://tcga‐data.nci.nih.gov/) and GEO database (https://www.ncbi.nlm.nih.gov).

## References

[1] Hartman RI , Lin JY . Cutaneous melanoma‐a review in detection, staging, and management. Hematol Oncol Clin North Am. 2019;33(1):25‐38. 30497675 10.1016/j.hoc.2018.09.005

[2] Ali Z , Yousaf N , Larkin J . Melanoma epidemiology, biology and prognosis. EJC Suppl. 2013;11(2):81‐91. 26217116 10.1016/j.ejcsup.2013.07.012 PMC4041476

[3] Dzwierzynski WW . Melanoma risk factors and prevention. Clin Plast Surg. 2021;48(4):543‐550. 34503715 10.1016/j.cps.2021.05.001

[4] Leonardi GC , Falzone L , Salemi R , et al. Cutaneous melanoma: from pathogenesis to therapy (review). Int J Oncol. 2018;52(4):1071‐1080. 29532857 10.3892/ijo.2018.4287 PMC5843392

[5] Turner N , Ware O , Bosenberg M . Genetics of metastasis: melanoma and other cancers. Clin Exp Metastasis. 2018;35(5–6):379‐391. 29722002 10.1007/s10585-018-9893-y

[6] Gowda R , Robertson BM , Iyer S , Barry J , Dinavahi SS , Robertson GP . The role of exosomes in metastasis and progression of melanoma. Cancer Treat Rev. 2020;85:101975. 32050108 10.1016/j.ctrv.2020.101975

[7] Sinha S , Singh SK , Jangde N , Ray R , Rai V . p32 promotes melanoma progression and metastasis by targeting EMT markers, Akt/PKB pathway, and tumor microenvironment. Cell Death Dis. 2021;12(11):1012. 34711805 10.1038/s41419-021-04311-5 PMC8553772

[8] Park SL , Buzzai A , Rautela J , et al. Tissue‐resident memory CD8(+) T cells promote melanoma‐immune equilibrium in skin. Nature. 2019;565(7739):366‐371. 30598548 10.1038/s41586-018-0812-9

[9] Zorov DB , Juhaszova M , Sollott SJ . Mitochondrial reactive oxygen species (ROS) and ROS‐induced ROS release. Physiol Rev. 2014;94(3):909‐950. 24987008 10.1152/physrev.00026.2013 PMC4101632

[10] Chiorcea‐Paquim AM . 8‐oxoguanine and 8‐oxodeoxyguanosine biomarkers of oxidative DNA damage: a review on HPLC‐ECD determination. Molecules. 2022;27(5):1620. 35268721 10.3390/molecules27051620 PMC8911600

[11] Aitken RJ , Smith TB , Jobling MS , Baker MA , De Iuliis GN . Oxidative stress and male reproductive health. Asian J Androl. 2014;16(1):31‐38. 24369131 10.4103/1008-682X.122203 PMC3901879

[12] Boiteux S , Coste F , Castaing B . Repair of 8‐oxo‐7,8‐dihydroguanine in prokaryotic and eukaryotic cells: properties and biological roles of the Fpg and OGG1 DNA N‐glycosylases. Free Radic Biol Med. 2017;107:179‐201. 27903453 10.1016/j.freeradbiomed.2016.11.042

[13] Visnes T , Benitez‐Buelga C , Cazares‐Korner A , et al. Targeting OGG1 arrests cancer cell proliferation by inducing replication stress. Nucleic Acids Res. 2020;48(21):12234‐12251. 33211885 10.1093/nar/gkaa1048 PMC7708037

[14] Zhao Z , Gad H , Benitez‐Buelga C , et al. NEIL3 prevents senescence in hepatocellular carcinoma by repairing oxidative lesions at telomeres during mitosis. Cancer Res. 2021;81(15):4079‐4093. 34045188 10.1158/0008-5472.CAN-20-1028 PMC9398161

[15] Gaillard H , Garcia‐Muse T , Aguilera A . Replication stress and cancer. Nat Rev Cancer. 2015;15(5):276‐289. 25907220 10.1038/nrc3916

[16] Macheret M , Halazonetis TD . DNA replication stress as a hallmark of cancer. Annu Rev Pathol. 2015;10:425‐448. 25621662 10.1146/annurev-pathol-012414-040424

[17] Zhao Z , He K , Zhang Y , et al. XRCC2 repairs mitochondrial DNA damage and fuels malignant behavior in hepatocellular carcinoma. Cancer Lett. 2021;512:1‐14. 33964350 10.1016/j.canlet.2021.04.026

[18] Belanger F , Angers JP , Fortier E , et al. Mutations in replicative stress response pathways are associated with S phase‐specific defects in nucleotide excision repair. J Biol Chem. 2016;291(2):522‐537. 26578521 10.1074/jbc.M115.685883 PMC4705374

[19] Lawaree E , Jankevicius G , Cooper C , Ahel I , Uphoff S , Tang CM . DNA ADP‐ribosylation stalls replication and is reversed by RecF‐mediated homologous recombination and nucleotide excision repair. Cell Rep. 2020;30(5):1373‐84 e4. 32023456 10.1016/j.celrep.2020.01.014 PMC7003065

[20] Flem‐Karlsen K , McFadden E , Omar N , et al. Targeting AXL and the DNA damage response pathway as a novel therapeutic strategy in melanoma. Mol Cancer Ther. 2020;19(3):895‐905. 31871265 10.1158/1535-7163.MCT-19-0290

[21] Kaushik Tiwari M , Adaku N , Peart N , Rogers FA . Triplex structures induce DNA double strand breaks via replication fork collapse in NER deficient cells. Nucleic Acids Res. 2016;44(16):7742‐7754. 27298253 10.1093/nar/gkw515 PMC5027492

[22] Romero OA , Sanchez‐Cespedes M . The SWI/SNF genetic blockade: effects in cell differentiation, cancer and developmental diseases. Oncogene. 2014;33(21):2681‐2689. 23752187 10.1038/onc.2013.227

[23] Schoenfeld AJ , Bandlamudi C , Lavery JA , et al. The genomic landscape of SMARCA4 alterations and associations with outcomes in patients with lung cancer. Clin Cancer Res. 2020;26(21):5701‐5708. 32709715 10.1158/1078-0432.CCR-20-1825 PMC7641983

[24] Yao B , Gui T , Zeng X , et al. PRMT1‐mediated H4R3me2a recruits SMARCA4 to promote colorectal cancer progression by enhancing EGFR signaling. Genome Med. 2021;13(1):58. 33853662 10.1186/s13073-021-00871-5 PMC8048298

[25] Li N , Li M , Hong W , et al. Brg1 regulates pro‐lipogenic transcription by modulating SREBP activity in hepatocytes. Biochim Biophys Acta Mol Basis Dis. 2018;1864(9 Pt B):2881‐2889. 29857051 10.1016/j.bbadis.2018.05.022

[26] Shi X , Wang Q , Gu J , Xuan Z , Wu JI . SMARCA4/Brg1 coordinates genetic and epigenetic networks underlying shh‐type medulloblastoma development. Oncogene. 2016;35(44):5746‐5758. 27065321 10.1038/onc.2016.108

[27] Husain A , Begum NA , Taniguchi T , Taniguchi H , Kobayashi M , Honjo T . Chromatin remodeller SMARCA4 recruits topoisomerase 1 and suppresses transcription‐associated genomic instability. Nat Commun. 2016;7:10549. 26842758 10.1038/ncomms10549 PMC4742980

[28] Chetty R , Serra S . SMARCA family of genes. J Clin Pathol. 2020;73(5):257‐260. 32312722 10.1136/jclinpath-2020-206451

[29] Bayona‐Feliu A , Barroso S , Munoz S , Aguilera A . The SWI/SNF chromatin remodeling complex helps resolve R‐loop‐mediated transcription‐replication conflicts. Nat Genet. 2021;53(7):1050‐1063. 33986538 10.1038/s41588-021-00867-2

[30] Coe EA , Tan JY , Shapiro M , et al. The MITF‐SOX10 regulated long non‐coding RNA DIRC3 is a melanoma tumour suppressor. PLoS Genet. 2019;15(12):e1008501. 31881017 10.1371/journal.pgen.1008501 PMC6934268

[31] Feng W , Liu S , Zhu R , et al. SOX10 induced nestin expression regulates cancer stem cell properties of TNBC cells. Biochem Biophys Res Commun. 2017;485(2):522‐528. 28189679 10.1016/j.bbrc.2017.02.014

[32] Wu Y , Fletcher M , Gu Z , et al. Glioblastoma epigenome profiling identifies SOX10 as a master regulator of molecular tumour subtype. Nat Commun. 2020;11(1):6434. 33339831 10.1038/s41467-020-20225-w PMC7749178

[33] Rosenbaum SR , Tiago M , Caksa S , et al. SOX10 requirement for melanoma tumor growth is due, in part, to immune‐mediated effects. Cell Rep. 2021;37(10):110085. 34879275 10.1016/j.celrep.2021.110085 PMC8720266

[34] Laurette P , Strub T , Koludrovic D , et al. Transcription factor MITF and remodeller BRG1 define chromatin organisation at regulatory elements in melanoma cells. Elife. 2015;4:e06857. 25803486 10.7554/eLife.06857 PMC4407272

[35] Marathe HG , Watkins‐Chow DE , Weider M , et al. BRG1 interacts with SOX10 to establish the melanocyte lineage and to promote differentiation. Nucleic Acids Res. 2017;45(11):6442‐6458. 28431046 10.1093/nar/gkx259 PMC5499657

[36] Weider M , Kuspert M , Bischof M , et al. Chromatin‐remodeling factor Brg1 is required for Schwann cell differentiation and myelination. Dev Cell. 2012;23(1):193‐201. 22814607 10.1016/j.devcel.2012.05.017

[37] Blakely AM , Cohen JT , Comissiong DS , Vezeridis MP , Miner TJ . Prognosis and Management of Thick and Ultrathick Melanoma. Am J Clin Oncol. 2019;42(11):824‐829. 31517636 10.1097/COC.0000000000000604

[38] Li C , Yin M , Wang LE , et al. Polymorphisms of nucleotide excision repair genes predict melanoma survival. J Invest Dermatol. 2013;133(7):1813‐1821. 23407396 10.1038/jid.2012.498 PMC3660504

