# Supplementary material for: SOX10 modulated SMARCA4 dysregulation alleviates DNA replication stress in cutaneous melanoma
Source: J Cell Mol Med. 2022 Nov 1;26(23):5846–57. doi: 10.1111/jcmm.17607 (PMC9716224; doi:10.1111/jcmm.17607)

**Supplementary Figures Legends**

Suppl. Figure 1.

A-B. Kaplan-Meier analysis comparing the disease-specific survival (A, DSS) and disease-free interval (B, DFI) in different clusters of melanoma patients from SC3 clustering.

C-E. Violin plot showing the distribution of age (C), Breslow depth value (D), and mitotic count rate (E) in Cluster 1-3 melanoma patients.

F. Percent of melanoma patients with different pathological staging in Cluster 1-3 melanoma patients.

G. Percent of patients undergoing radiation therapy, pharmaceutical therapy, neoadjuvant treatment, or additional surgery in Cluster 1-3 melanoma patients.

H-I. Distribution of different Clark value levels (H) or types of new tumor events (I) in Cluster 1-3 melanoma patients.

Suppl. Figure 2.

A. Heatmap showing the expression of all Cluster 3 marker genes in melanoma patients from TCGA SKCM database.

B. Heatmap showing the correlation between all Cluster 3 marker genes. And genes with Pearson correlation > 0.3 with at least 50% of other genes were labeled in red.

C-D. Dot plot showing the disease-specific survival (C, DSS) or disease-free interval (D, DFI) was shown with the expression of Cluster 3 genes.

E. Volcano plot showing the expression level of all feature genes in Cluster 3 vs. Cluster 1/2. p value (Log_10_p value) and the fold change (Log_2_FC) were calculated with the student’s *t-test*. The upregulated genes with Log_2_FC>0.5 and Log_2_p value>10 were labeled in red.

F. Multivariate Cox regression result for the disease-specific survival (DSS) in melanoma patients in TCGA SKCM database.

Suppl. Figure 3.

A. Kaplan-Meier analysis comparing the disease-specific survival (DSS, the upper panel) or the disease-free interval (DFI) with different SMARCA4, NUDT1, or XBA2 levels.

B-E. Violin plot comparing the expression of SMARCA4 with different BRAF status (B, GSE50509 database), pathological staging (C, TCGA database), Clark level value (D, TCGA database), and metastatic condition (E, TCGA database).

F. Dot plot showing the correlation between Breslow depth value and SMARCA4 mRNA level. Pearson correlation was performed and the p value was calculated.

Suppl. Figure 4.

A. Kaplan-Meier analysis comparing the overall survival (OS), disease-specific survival (DSS), or disease-free interval (DFI) in mutated or wild-type SMARCA4 patients.

B. Kaplan-Meier analysis comparing the overall survival (OS), disease-specific survival (DSS), or disease-free interval (DFI) with different SMARCA4 copy number variations.

C. Quantification of flow cytometry data comparing the relative percentage of necrotic cells (Annexin-V+, PI+) and early apoptotic cells (Annexin-V+, PI-) in SMARCA4 depleted cells.

Suppl. Figure 5.

A. Violin plot showing the relative methylation status of SMARCA4 gene in TCGA database.

B. Table showing the Pearson correlation results.

C. Kaplan-Meier analysis comparing the overall survival (OS) of melanoma patients with different levels of methylation at different sites of the SMARCA4 genes. cg08151828 (the left panel), cg26967868 (the middle panel) and cg26967868 (the right panel) were analyzed respectively.

D. Dot plot showing the correlation between SMARCA4 mRNA and cg08151828 (the left panel), cg26967868 (the middle panel), and cg26967868 (the right panel).

E. Validation of GAPDH as stable qPCR control in A375 (the right panel) and M14 (the left panel) cells. 50ng of cDNA was used and the CT value for GAPDH and ACTB were measured with qPCR in SMARCA4 depleted M14 and A375 cells.

**Supplementary Figures**

Suppl. Figure 1.


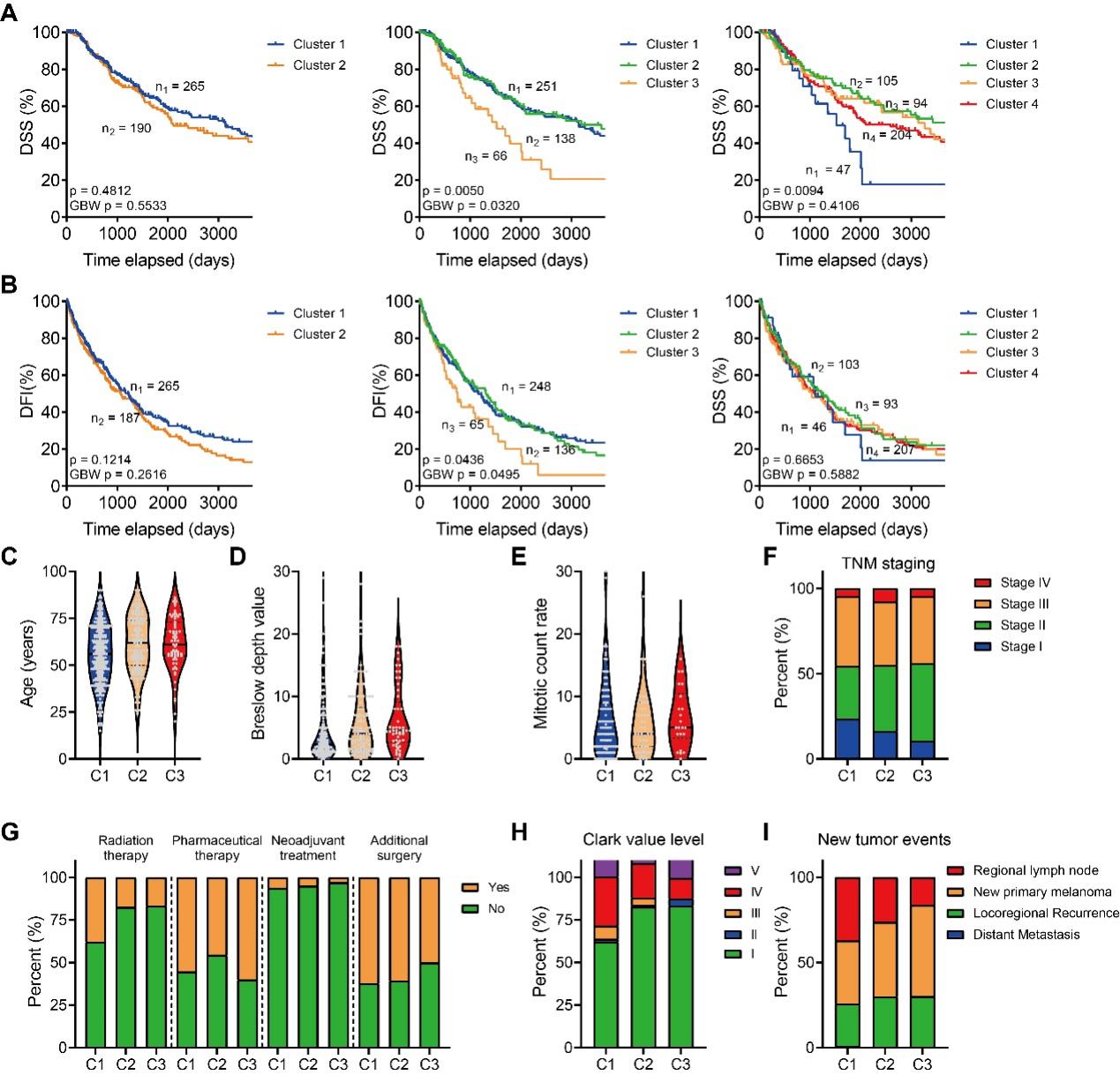


Suppl. Figure 2.


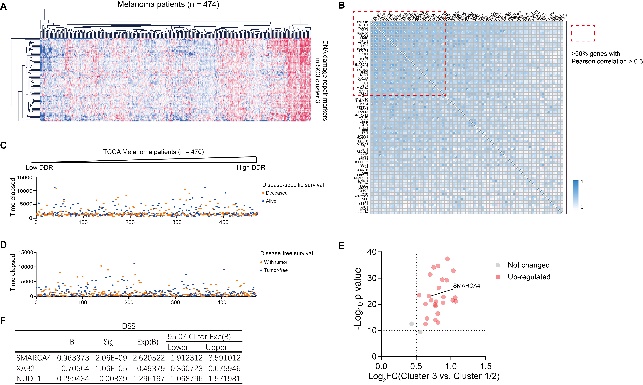


Suppl. Figure 3.


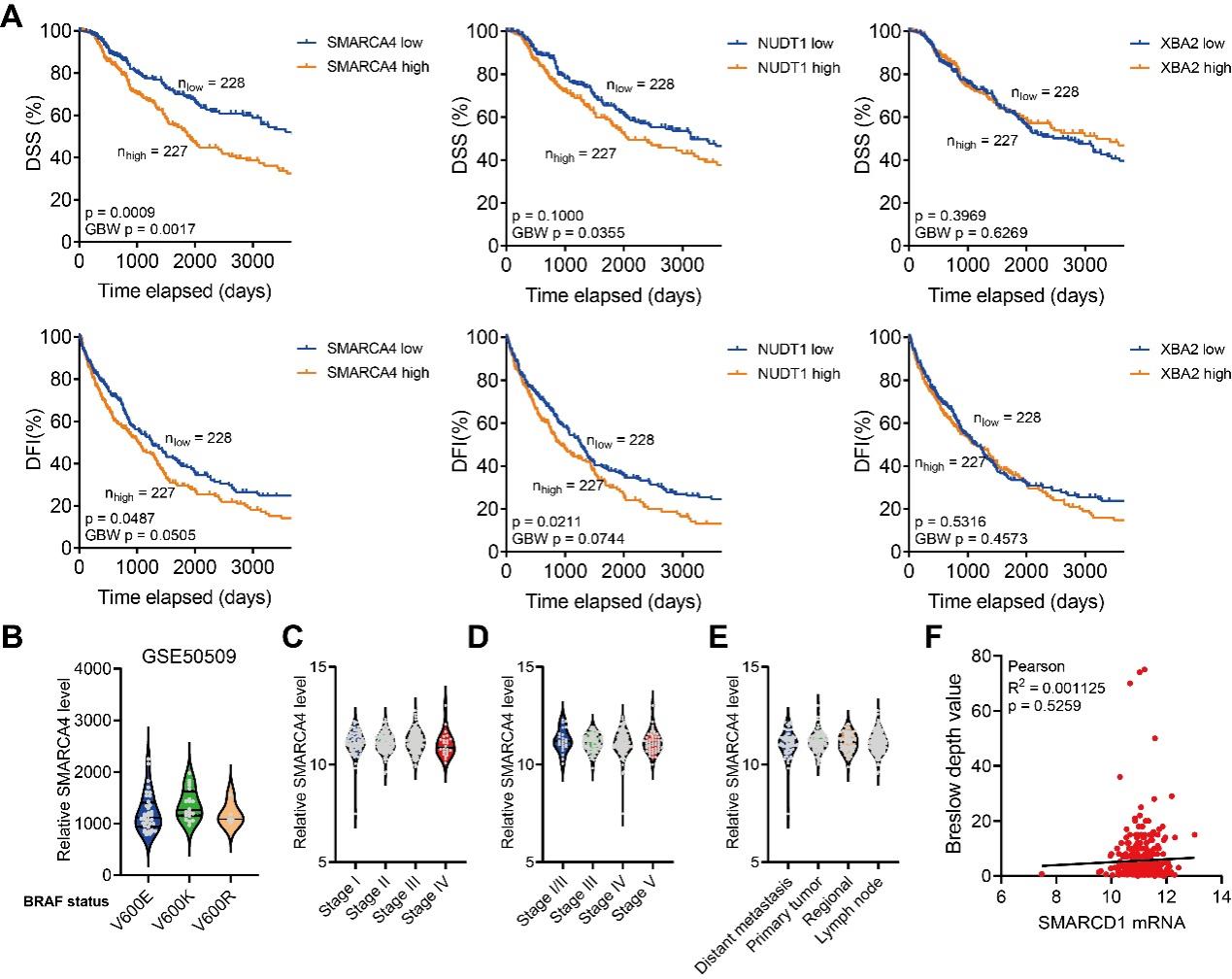


Suppl. Figure 4.


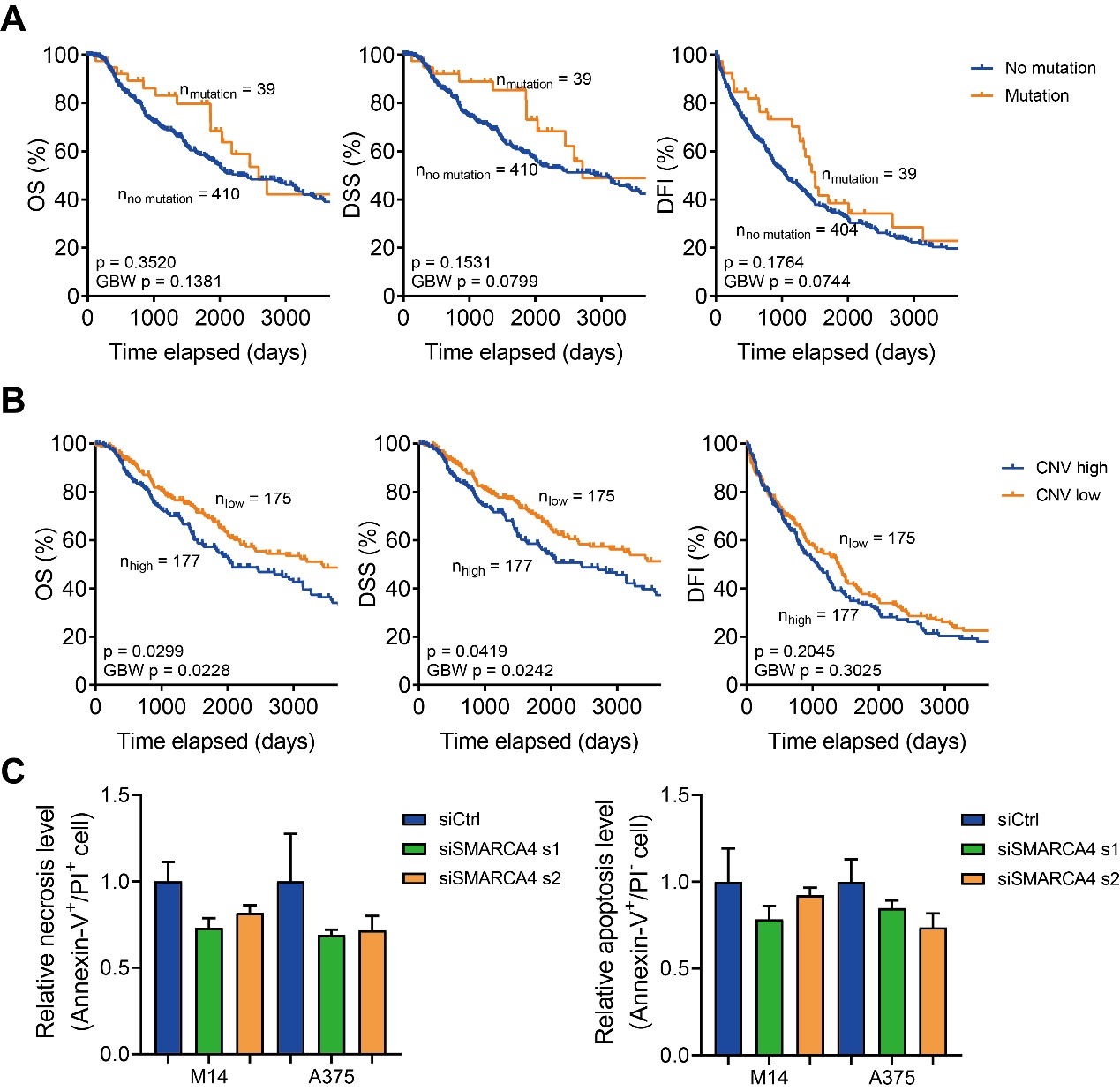


Suppl. Figure 5.


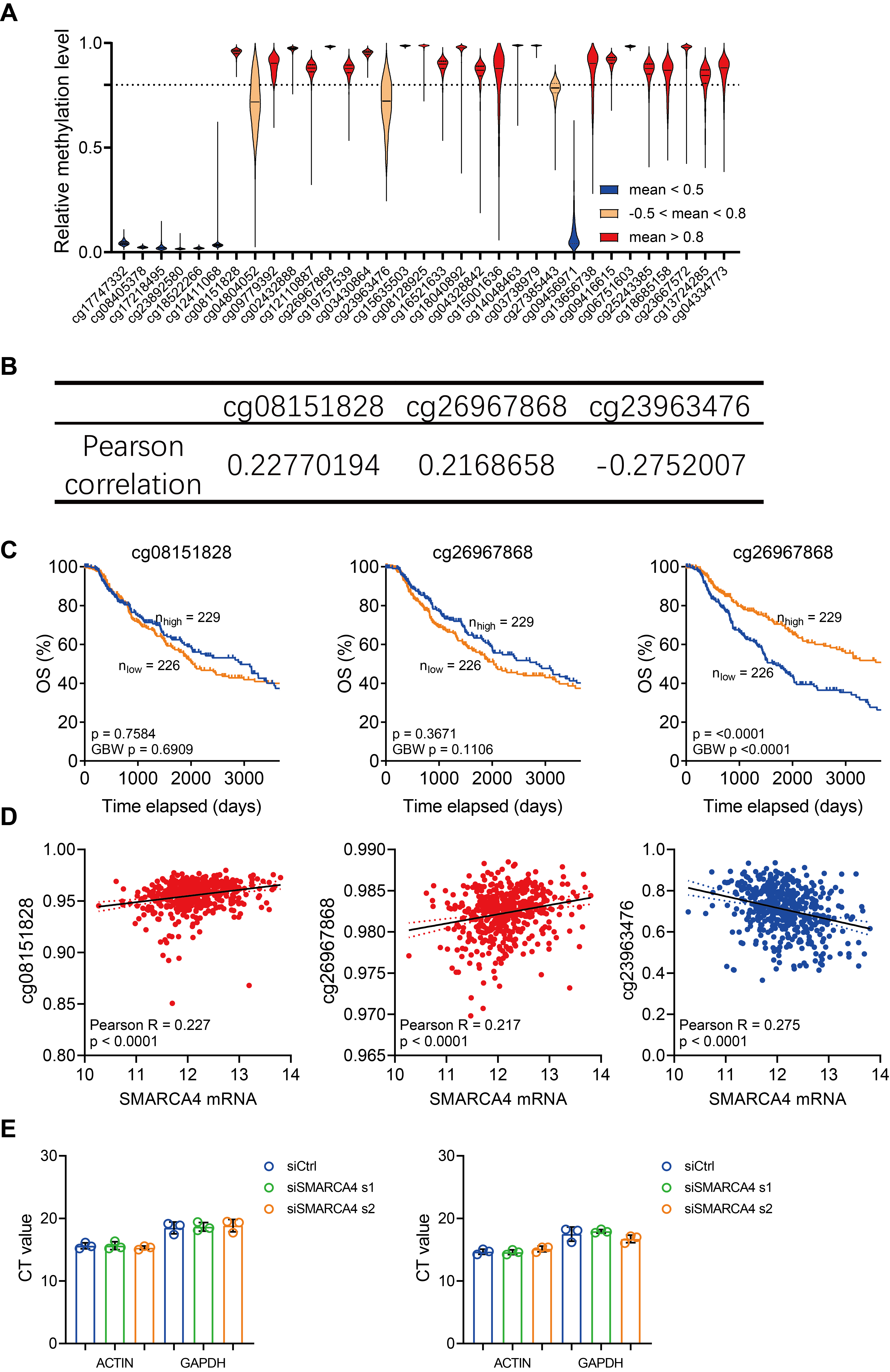

Supplement: Supplementary file 1 — Figure S1 [file JCMM-26-5846-s001.docx]
